# nPhase: an accurate and contiguous phasing method for polyploids

**DOI:** 10.1186/s13059-021-02342-x

**Published:** 2021-04-29

**Authors:** Omar Abou Saada, Andreas Tsouris, Chris Eberlein, Anne Friedrich, Joseph Schacherer

**Affiliations:** 1 Université de Strasbourg, CNRS, GMGM UMR, 7156 Strasbourg, France; 2 Institut Universitaire de France (IUF), Paris, France

**Keywords:** Polyploid, Haplotype, Phasing, Long-read sequencing, Pipeline

## Abstract

While genome sequencing and assembly are now routine, we do not have a full, precise picture of polyploid genomes. No existing polyploid phasing method provides accurate and contiguous haplotype predictions. We developed nPhase, a ploidy agnostic tool that leverages long reads and accurate short reads to solve alignment-based phasing for samples of unspecified ploidy (https://github.com/OmarOakheart/nPhase). nPhase is validated by tests on simulated and real polyploids. nPhase obtains on average over 95% accuracy and a contiguous 1.25 haplotigs per haplotype to cover more than 90% of each chromosome (heterozygosity rate ≥ 0.5%). nPhase allows population genomics and hybrid studies of polyploids.

**Supplementary Information:**

The online version contains supplementary material available at 10.1186/s13059-021-02342-x.

## Background

Studying genotype-phenotype relations is contingent on having an accurate view of the genetic variants. To that end, various sequencing strategies and ways to analyze them have been developed. The ultimate goal is to faithfully determine the precise sequence of the DNA molecules contained within the cell. In practice, this level of precision is rarely necessary and approximations are routinely used when they can be afforded. Aligning the sequencing data to a reference genome is a good approximation to identify genetic variants such as single nucleotide polymorphisms (SNPs) but a poor one to identify structural variants (SVs) [1]. By contrast, the generation of de novo assemblies using the sequencing data is a good approximation to identify SVs [1] but, without significant polishing work [2], usually leads to a lower quality sequence. One enduring approximation is the reduction of the genome to a single sequence, even if the organism does not have a haploid or rigorously homozygous genome. A diploid or higher ploidy genome can be heterozygous. Identifying the heterozygous positions, or variants, is known as genotyping. Linking these variants together to establish which variants co-occur on the same strand of DNA is known as haplotyping or phasing. There is increasing interest in phasing genomes for diverse reasons, such as to obtain more accurate reference genomes [3], better study population genomics [4], improve the accuracy of GWAS studies [5], study the effects of compound heterozygosity [6], investigate Allele-Specific Expression patterns [7], gain insight into polyploid evolution [8, 9], better understand the mechanisms of heterosis [10], and dissect the origins of hybrid species [11].

Phased genomes can be obtained either by physically separating entire chromosomes [12] (or significantly large portions of chromosomes) prior to sequencing [13] or by separating them bioinformatically after sequencing the whole genome [14]. The length of reads is a significant limiting factor in the ability to bioinformatically separate reads into their corresponding haplotypes. One very successful method that overcame that limitation was trio binning [15], which circumvented the importance of long reads by leveraging information from parental whole genome sequencing. Other methods have been explored but cannot overcome the short-read length limitation particularly well [16]. One solution has been to resort to imputing haplotypes through reference panels [17]. Despite a higher error rate, diploid phasing of long reads has been solved by existing methods such as WhatsHap [18], an alignment-based phasing tool, and Falcon-Unzip [19], an assembly-based phasing tool. Assembly-based phasing attempts to generate a de novo assembly for each haplotype directly, without relying on a reference sequence. Alignment-based phasing uses a reference genome as support to identify heterozygous positions and then attempts to link positions together based on the co-occurrence of heterozygous SNPs on overlapping reads. For diploids, each variable position can only be one of two possible bases. Knowing one haplotype allows to deduce the other. This allows diploid phasing methods to be relatively simple and straight-forward. For polyploids, however, a variable position can be one of two or up to six possible states (all four bases, a deletion or an insertion) and this deduction is no longer possible, rendering the task of phasing significantly more complex. Some methods currently exist to phase polyploids but mainly using short-read sequencing and leading to a low accuracy and contiguity phasing [20–23].

Here, we developed nPhase to address the lack of a polyploid phasing method that outputs accurate, contiguous results and does not require prior knowledge of the ploidy of the sequenced genome. The required inputs are a reference sequence as well as long- and short-read sequencing data. The pipeline performs the mapping, variant calling, and phasing and outputs the phased variants and a fastQ file for each predicted phased haplotype, or haplotig. The nPhase algorithm is ploidy agnostic, meaning it does not require any prior knowledge of ploidy and will not attempt to guess the ploidy of the sample. Instead, it will separate the reads into as few distinct haplotigs as possible. The nPhase algorithm has three modifiable parameters; we have evaluated the effects of these parameters on the results and provide a default set of parameters, which we predict to be appropriate for all cases, along with some recommendations on how to modify these parameters for genomes that are more difficult to phase, i.e., low heterozygosity and high ploidy genomes.

Using yeast as an in silico model, we validated the performance of nPhase on simulated *Saccharomyces cerevisiae* genomes (2n, 3n, and 4n) of varying heterozygosity levels (0.01%, 0.05%, 0.1%, and 0.5% of the genome) as well as on a triploid *Brettanomyces bruxellensis* sample and chromosome 2 of the autotetraploid potato plant, *Solanum tuberosum*. Based on our simulated tests, we found that nPhase performs very well in terms of accuracy and contiguity. We obtained an average of 93.9% accuracy for all diploids, 92.3% for all triploids, and 94.5% for tetraploids with a heterozygosity level of at least 0.5%, or 87.3% accuracy when we include the lowest heterozygosity level tetraploids. All results are very contiguous, with an average of between 2.4 and 4.1 haplotigs per haplotype, bringing us very close to the ideal result of one haplotig per haplotype.

## Results

### Phasing pipeline and strategy

We developed the nPhase pipeline, an alignment-based phasing method and associated algorithm that run using three inputs: highly accurate short reads, informative long reads, and a reference sequence. The pipeline takes the raw inputs and processes them into data usable by the nPhase algorithm. Unlike other existing methods, our algorithm is designed for ploidy agnostic phasing. It does not require the user to input a ploidy level and it does not contain any logic that attempts to estimate the ploidy of the input data. The idea at the core of the algorithm is that if you iteratively cluster the most similar long reads and groups of long reads together, you will naturally recreate the original haplotypes.

**Fig. 1:**
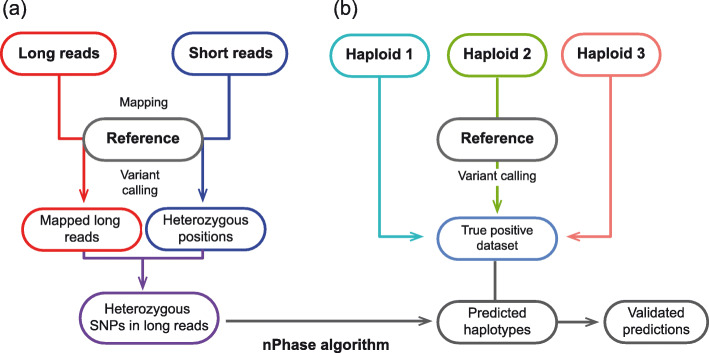
nPhase pipeline and verification process. **a** The nPhase pipeline requires three inputs: a long-read dataset, a short-read dataset, and a reference genome sequence. Both sequencing datasets are mapped to this reference genome; then, the short reads are variant called in order to identify heterozygous positions. The long reads are reduced to only their heterozygous positions, and this set of linked heterozygous positions is phased by the nPhase algorithm and outputs phased haplotypes. **b** In parallel with running the virtual polyploids through the nPhase pipeline, we map the original strains to the same reference and variant call them to identify their haplotypes. This generates the true-positive dataset against which we will compare the haplotypes predicted by nPhase in order to assess the accuracy of our algorithm

For the first step of the pipeline, the long and short reads are aligned to the reference, then the aligned short reads are variant called to identify heterozygous positions and generate a high-quality dataset of variable positions (Fig. 1). Each long read is then reduced to its set of heterozygous SNPs according to the previously identified variable positions. We also collect long-read coverage information to allow the level of representation of a haplotype in the data to influence its likelihood of being properly phased (see the “Methods” section).

The reduced long reads and the coverage information are then passed onto the nPhase algorithm, an iterative clustering method. On the first iteration, nPhase clusters together the two most similar long reads, then it checks that the cluster identities are maintained, i.e., it checks that merging these two long reads together does not significantly change the information they each contain individually, and finally, it generates a consensus sequence representative of the group of these two reads. The next iteration will be exactly the same with N^− 1^ reads. nPhase will run until all remaining clusters are sufficiently different from each other to fail the cluster identity maintenance check. These remaining clusters represent the different haplotypes within the original dataset.

### nPhase, a ploidy agnostic phasing algorithm

**Fig. 2:**
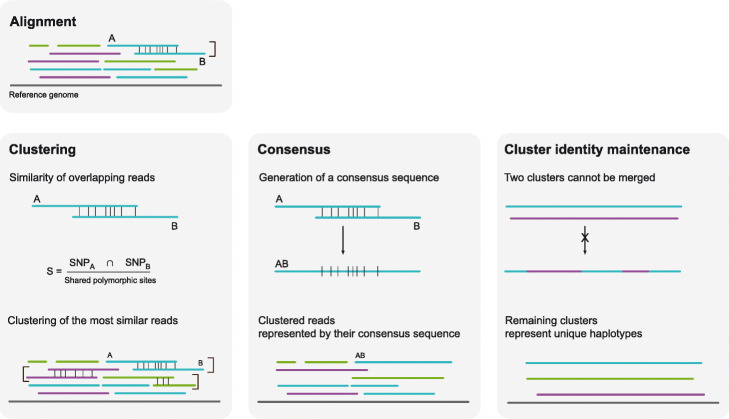
nPhase algorithm. Here we represent how a triploid’s reads could align to a reference sequence. Each read is one of three colors, one for each haplotype. The clustering, consensus, and cluster identity maintenance steps are iteratively repeated until all remaining clusters are forbidden to merge. Clustering: each vertical line represents a SNP; different colors signify different haplotypic origins. Only two reads are clustered at a time; here we show three clusters, so this is the result of the third step of nPhase’s iterative clustering. Consensus: a consensus sequence is generated by allowing every read in the cluster to vote for a specific base for a given position. Votes are weighted by the pre-calculated context coverage number to discourage sequencing errors. The consensus sequences that represent clusters are treated just like aligned long reads and continue to be clustered. Cluster identity maintenance: when all remaining clusters are very different from each other, they are not allowed to merge; this is to prevent the algorithm from always outputting only one cluster per region. The remaining clusters and their consensus sequences should correspond to the haplotypes present in the original dataset

As described earlier, nPhase is an iterative clustering algorithm. It is composed of three main ideas: (i) clustering, which ensures that similar reads are clustered together; (ii) cluster identity maintenance, which ensures that only similar clusters are merged into larger ones; and finally (iii) consensus, a way to reduce a cluster to a consensus sequence in order to easily compare it to other clusters (Fig. 2).

Each step of the clustering algorithm starts by calculating the similarity between every overlapping pair of reads (Fig. 2a). By default, the minimal overlap is 10 heterozygous positions. Similarity is defined as *S* = *N*_shared variants_/*N*_shared positions_. The pair of reads with the highest similarity is clustered together. If there is a tie, then we cluster together the pair of reads with the most variable positions in common. If there is again a tie, then we select a pair randomly. By default, the algorithm will not attempt to merge two sequences with less than 1% similarity.

The pair that was selected now forms a cluster of two reads (Fig. 2b). In order to continue this iterative algorithm, we need to define a way to calculate the similarity between a read and a cluster of reads, and the similarity between two clusters of reads. We do so by computing a consensus sequence for each cluster of reads and we use the consensus sequence to calculate the similarity as defined above. For each position, the consensus is defined as the base which has the most support from the reads in the cluster. Each read gets a vote equal to the context coverage of the base it supports. If there is a tie, then all tied bases are included in the consensus sequence.

As defined, the clustering algorithm will continue to iterate, merging clusters together until all available options are exhausted and output only one cluster per region (Fig. 2c). The solution is to set restrictions on which clusters are allowed to be merged in the clustering step. We consider that each cluster has its own “identity” defined by the population of reads that comprise it. If merging two clusters has a significant effect on the identity of both clusters, then the merge is not allowed. We calculate how much merging of two clusters would change them. The amount of change allowed is limited by the ID parameter. In order to quantify the amount of change to a cluster’s identity, we keep track of the “demographics” of each position, i.e., how strongly represented each base is for that position in that cluster. We differentiate positive identity changes from negative identity changes: (i) if a merge of two clusters results in increased support for their consensus sequence bases, then that change is considered positive; (ii) if the merge results in decreased support for a consensus sequence base, then that change is considered negative; and we calculate how many votes the base lost, even if it remains the consensus base after the merge. The number of votes lost is divided by the total number of votes in the region that both clusters have in common to obtain the cluster identity change percentage. By default, if it is higher than 5% we do not allow the two clusters to merge. Once all remaining clusters fail this test, the algorithm stops. The resulting clusters represent the different haplotypes that nPhase found and are output as different sets of reads, heterozygous SNPs, and consensus sequences.

### Validation of the nPhase algorithm by combining reads of non-heterozygous individuals

To test and validate the performance of nPhase, we decided to combine sequencing datasets of haploid and homozygous diploid organisms into virtual polyploid datasets. We selected four natural *S. cerevisiae* isolates as the basis for our virtual genomes: ACA, a haploid strain, and three homozygous diploid strains: CCN, BMB and CRL (Additional file 1: Table S1). These four strains have different ecological and geographical origins and are sufficiently distinct from each other to allow us to evaluate the performance of nPhase at heterozygosity levels of up to 1% of the genome [24].

We sequenced these strains using an Oxford Nanopore long-read sequencing strategy and obtained Illumina short-read data from our 1011 yeast genomes project [24]. Since these strains do not have any heterozygosity, we could map their short reads to the *Saccharomyces cerevisiae* reference genome and variant call them to obtain their haplotypes (Fig. 1). We then used these haplotypes as a truth set to assess the performance of nPhase. With this truth set, we tested the influence of dataset characteristics: coverage, ploidy, heterozygosity level, and the inclusion or exclusion of reads that map to distant regions of the genome, hereafter described as split reads. We also investigated the influence of parameters that modulate the behavior of the nPhase algorithm: minimum similarity, minimum overlap, and maximum ID change (for a description of them, see the available parameters in the “Methods” section).

To assess the influence of ploidy, we used the three constructions of the different virtual genomes previously mentioned. We also randomly sampled 6250, 12,500, 62,500, and 125,000 heterozygous SNPs from each virtual genome to simulate datasets where 0.05%, 0.1%, 0.5%, and 1% of the positions in the genome are heterozygous. This equates to three different ploidies and four heterozygosity levels, or 12 polyploid genomes to test.

By running a total of 6000 validation tests on varying ploidy, heterozygosity, and coverage levels exploring the parameter space, we determined default parameters of nPhase (see the “Methods” section). According to these tests, the parameters that result in optimal results in terms of accuracy and contiguity are the following: 1% minimum similarity, 10% minimum overlap, and 5% maximum ID (see the “Identifying optimal parameters” section in the “Methods” section). We then ran nPhase with these default parameters on our previously described optimal datasets of varying ploidy (2n, 3n, and 4n) and heterozygosity levels (0.05%, 0.1%, 0.5% and 1%) of 20X long reads per haplotype with split read information (Additional file 1: Table S2).

**Fig. 3:**
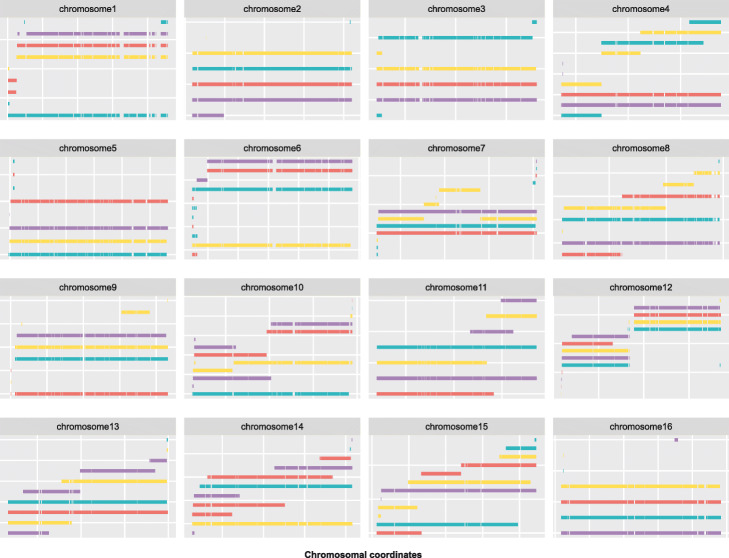
Predicted haplotypes for the tetraploid genome with a 0.5% heterozygosity level. The result of this test was an accuracy of 93.7%, an error rate of 4.0%, and a missing rate of 2.2% with an average of 2.4 haplotigs per haplotype. Each subgraph displays the predicted haplotigs for a different chromosome, each predicted haplotig is on a different row on the *Y* axis, and the *X* axis displays the position along the chromosome. All predicted haplotigs are color coded according to the haplotype they are the closest to

As an example, we phased the tetraploid genome showing a heterozygosity level of 0.5% using nPhase (Fig. 3). Since we know the ground truth, we can assign each haplotig to the strain whose haplotype it most closely represents and we can calculate our accuracy metrics.

In order to measure accuracy, we distinguish between two forms of errors: standard errors, i.e., heterozygous SNPs erroneously attributed to the wrong haplotype, and missing errors, i.e., heterozygous SNPs which we know are present but which were erroneously not represented in the predictions. The accuracy is the percentage of all SNPs which were correctly attributed to their haplotype. The error rate is the percentage of all predictions which were incorrect. The missing rate is the percentage of all heterozygous SNPs which were never attributed to their haplotype. We use the following formula: accuracy = TP/(TP + FP + FN) with TP = true positive; the SNP was attributed to the correct haplotype.

FP=false positive; the SNP does not belong in this haplotype.

FN=false negative; the SNP is not represented in the results.

The result of this test was an accuracy of 93.7%, an error rate of 4.0%, and a missing rate of 2.2% with an average of 2.4 haplotigs per haplotype. Seven of the sixteen chromosomes have an L90 of 1, meaning that for all four haplotypes, more than 90% of the heterozygous SNPs were assigned to one haplotig. For the nine remaining chromosomes, seven have at least two chromosome-length haplotigs. In all cases, the chromosomes are nearly fully covered by haplotigs that represent the four different haplotypes, as confirmed by the low missing haplotype prediction rate (2.2%). As a ploidy agnostic tool, nPhase was not given any information about the ploidy of this sample and does not attempt to estimate its ploidy. Despite that, nPhase reached a high accuracy (93.7%) and contiguity (2.4 haplotigs per haplotype), demonstrating its ability to reliably phase a tetraploid of that heterozygosity level. The same representation is available for the other datasets of different ploidy and heterozygosity levels (Additional file 2: Fig. S1).

**Fig. 4:**
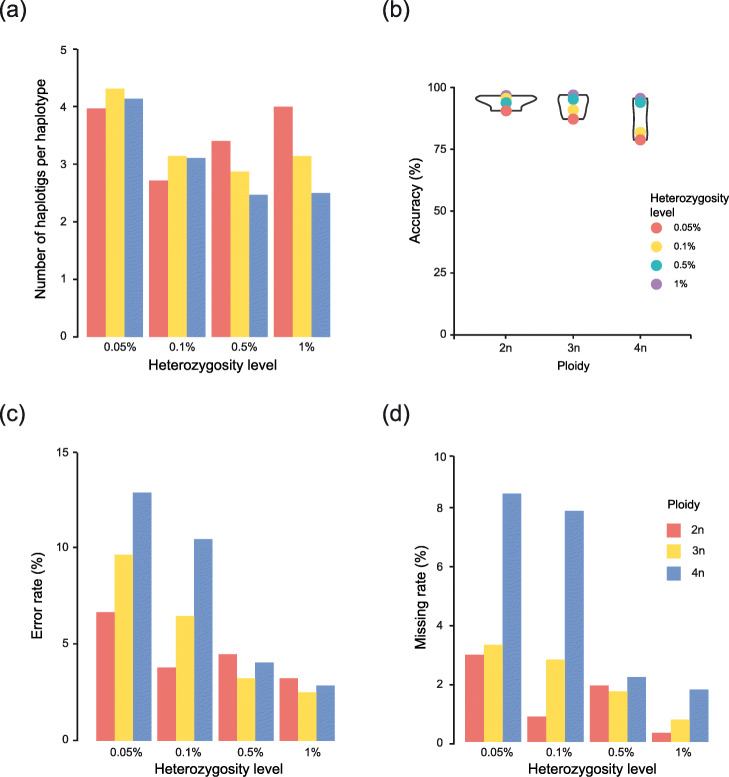
Effects of ploidy and heterozygosity levels on accuracy and contiguity. Through these graphs, we show the effects of sample properties (heterozygosity level and ploidy) on nPhase’s accuracy metrics when run with default parameters. **a** Each bar displays the contiguity of a different test result. The least contiguous heterozygosity level is 0.05%, likely related to its also yielding the least accurate results. Overall, we note little absolute variation in the contiguity. Interestingly, contiguity at higher heterozygosity levels appears to be a function of ploidy. Higher ploidies seem less likely to become less contiguous as a result of increasing the heterozygosity level, while the diploid tests are more affected. We also note that tetraploids of high heterozygosity level are the most contiguous. **b** Each bar displays the accuracy of a different test result. As ploidy increases, the accuracy tends to decrease. It also appears to decrease faster for tests on low heterozygosity level constructions. **c**, **d** Each bar displays our evaluation of the effects of ploidy and heterozygosity level on the error and missing rates, respectively, for our 12 tests using optimal parameters. Overall, we see that the error rate is always higher than the missing rate across these conditions. As the heterozygosity level increases, the error and missing rates decrease along with the gap between ploidies. We also find that more difficult phasing problems (high ploidy and low heterozygosity level) yield much higher error and missing rates and that the low heterozygosity tetraploids seem to be particularly sensitive to missing calls

Across the 12 phased genomes with variable ploidy and heterozygosity levels, we noted little variation in terms of contiguity as we obtained between 2.4 and 4.3 haplotigs per haplotype (Fig. 4a). At a heterozygosity level of 0.05%, the least contiguous genomes are observed with around 4 haplotigs per haplotype (Fig. 4a). The triploid genomes decrease to around 3 haplotigs per haplotype for heterozygosity levels greater than 0.1% (Fig. 4a). The tetraploid tests continue the trend of higher ploidies becoming more stable and contiguous as the heterozygosity level increases, dropping to 3.1 haplotigs per haplotype at the 0.1% heterozygosity level and then stabilizing at 2.4 haplotigs per haplotype at the 0.5% and 1% heterozygosity levels (Fig. 4a). This could be explained by the availability of more haplotigs to potentially merge with each other as ploidy increases.

Regarding the accuracy, we observed that for heterozygosity levels greater than 0.5%, the accuracy appears stable and high across ploidies with a minimum of 93.56% for the diploid (2n) at a 0.5% heterozygosity level, and a maximum of 96.70% accuracy for the triploid (3n) at a 1% heterozygosity level (Fig. 4b). For lower heterozygosity levels (≤ 0.1%), we have results that are more variable between ploidies (Fig. 4b). Diploid tests retain a high 95.32% accuracy for the 0.1% heterozygosity level but drop to 90.34% accuracy for the 0.05% heterozygosity level. For triploid genomes, the results drop to 90.70% accuracy for the 0.1% heterozygosity level, then down to 87.00% at 0.05% heterozygosity level. Continuing the trend of higher ploidies performing worse with lower heterozygosity levels, the accuracies for the 0.1% and 0.05% heterozygosity levels for the tetraploid tests output 81.65% and 78.62% accuracy, respectively.

In addition, we observed that errors are more frequent in all tests than missing calls (Fig. 4c, d). For higher heterozygosity levels (≥ 0.5%), these two forms of error are stable and very low. The error rate is set between a minimum of 2.53% for the 1% heterozygosity level triploid and a maximum of 4.51% for the 0.5% heterozygosity level diploid. And the missing rate is set between a minimum of 0.31% for the 1% heterozygosity level diploid and a maximum of 2.21% for the 0.5% heterozygosity level tetraploid. For lower heterozygosity levels (≤ 0.1%), both the error and missing rates increase with ploidy, suggesting both types of errors may be linked. If we set aside the 0.1% heterozygosity level diploid which has an error and missing rates of 3.82% and 0.86%, respectively, the error rates have a wide range with a minimum error of 6.49% for the 0.1% heterozygosity level triploid and a maximum error of 12.91% for the 0.05% heterozygosity level tetraploid. Similarly, the missing rates range from a minimum of 2.97% for the 0.05% heterozygosity level diploid to a maximum of 8.46% for the 0.05% heterozygosity level tetraploid, again adding to the trend of lower heterozygosity levels coupled with higher ploidies yielding worse results.

### Benchmarking nPhase against other polyploid phasing tools

Some methods currently exist to phase polyploids using long-read data such as WhatsHap polyphase [20], as well as other methods which were mostly designed to work with short-read sequencing data but can sometimes use long reads as input [21–23]. Because nPhase is a phasing tool that leverages the linking power of long reads to achieve its high accuracy and contiguity metrics, we did not benchmark it against tools that rely exclusively on short reads for phasing, since these are inherently limited by the size of their reads. We also did not benchmark nPhase against tools that can only phase diploid genomes as this is not the intended use case for our algorithm. We therefore compare nPhase to the recently released WhatsHap polyphase, to our knowledge the only other polyploid phasing algorithm that handles long reads.

**Fig. 5:**
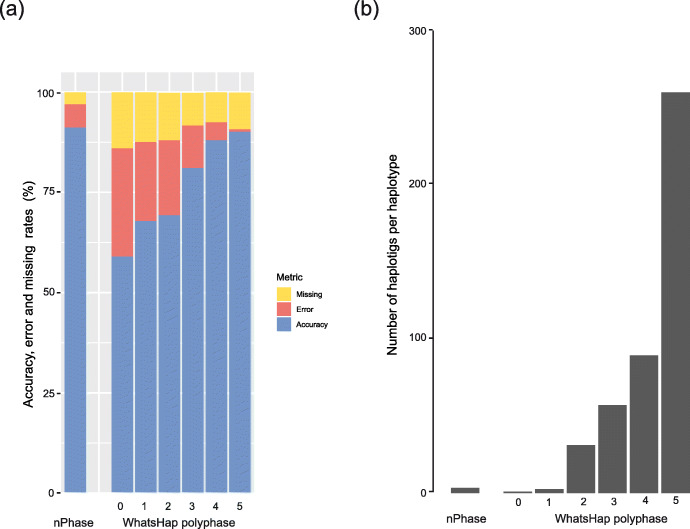
Error types and number of haplotigs for nPhase and WhatsHap polyphase. nPhase and WhatsHap polyphase were both applied to our 20X test datasets of different ploidy and heterozygosity levels. nPhase was tested using its default parameters and WhatsHap polyphase was tested with all six possible values of its adjustable sensitivity parameter. This graph compares both tools using the following metrics: average number of haplotigs obtained for the genome, normalized by the ploidy, average accuracy, average error rate, and average missing rate. **a** Average accuracy, error, and missing rates for all tests using nPhase and WhatsHap polyphase on different sensitivity levels. The error rate for WhatsHap polyphase increases dramatically as the sensitivity level decreases, illustrating the tool’s tradeoff between accuracy and contiguity. **b** Average number of haplotigs per chromosome per haplotype for all tests using nPhase and WhatsHap polyphase on different sensitivity levels. The very high number of haplotigs per chromosome per haplotype for the highest sensitivity levels (5 and 4) shows that despite being highly accurate, they are not contiguous enough to be informative. Based on our results, nPhase outperforms WhatsHap polyphase in all of our tests. The tradeoff between accuracy and contiguity is extreme in WhatsHap polyphase, either the results are very accurate but so fragmented as to be uninformative, or they are about as contiguous as nPhase but less than 65% accurate

We compared the results nPhase (default parameters) with WhatsHap polyphase on the same samples (Fig. 5). Since WhatsHap polyphase has a parameter named “--block-cut-sensitivity” that can be set to determine the tradeoff between accuracy and contiguity, we tested WhatsHap polyphase using all possible values for this parameter (integers from 0 to 5) to compare all possible results to nPhase’s default results. A value of 0 for this parameter means that WhatsHap polyphase will generate the most contiguous results possible, and 5 means that it will generate the most accurate results possible.

The performance of WhatsHap polyphase was measured in terms of switch error rate and N50 block lengths. Instead, we will talk about accuracy and average number of haplotigs per haplotype, two metrics that are more direct representations of the performance of the algorithms and answer two important questions: “How reliable are the results?”, i.e., what are the proportions of accurate, erroneous and missing calls? And “How informative are they?”, i.e., by how many haplotigs is each haplotype represented? nPhase and WhatsHap polyphase were both applied to our 20X test datasets of different ploidy and heterozygosity levels. nPhase was tested using its default parameters and WhatsHap polyphase was tested with all six possible values of its adjustable sensitivity parameter. We report here the average accuracy, error, and missing rates, as well as the average number of haplotigs obtained for the genome, normalized by the ploidy.

In our tests, nPhase has an average accuracy of 91.2%, slightly outperforming WhatsHap polyphase’s most sensitive setting (5), which yields an average accuracy of 90.1%, and its second most sensitive setting (4) which yields an average accuracy of 88.9% (Fig. 5a). Lower sensitivity levels for WhatsHap polyphase quickly lose accuracy, with the next lowest setting yielding only 81.1% accuracy on average, and its least sensitive setting only reaching 59% accuracy.

In addition to its high accuracy, nPhase is highly contiguous, outputting these accurate results, on average, in 3.4 haplotigs per chromosome per haplotype (Fig. 5b). The highly accurate WhatsHap polyphase sensitivity levels (5 and 4) output their results in a highly discontiguous 258.7 and 88.9 haplotigs per haplotype, respectively. In order to output results of similar contiguity to nPhase, WhatsHap polyphase must sacrifice accuracy and drop to a sensitivity level of 1 or 0, which output 2.5 and 0.9 haplotigs per chromosome per haplotype, respectively. This tradeoff between accuracy and contiguity performed by WhatsHap polyphase does not appear to have a useful middle ground and nPhase demonstrates that it is not necessary to make a choice given that it simultaneously achieves both.

### Validation of the nPhase algorithm on a real *Brettanomyces bruxellensis* triploid strain

**Fig. 6:**
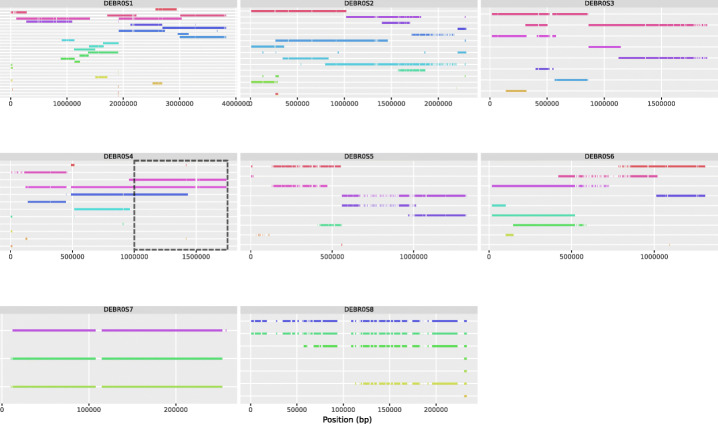
Predicted haplotypes for the *Brettanomyces bruxellensis* strain. Each subgraph displays the predicted haplotigs for a different chromosome of this 0.7% heterozygosity level triploid, each predicted haplotig is on a different row on the Y axis, and the X axis displays the position along the chromosome. All predicted haplotigs are color coded randomly as the ground truth is not known. We observe that while the strain is a known triploid, nPhase did not exclusively predict three haplotypes per region. We also note that some regions such as the end of chromosome 2 or center of chromosome 6 have a very low level of heterozygosity

We further tested nPhase by running it on a real triploid organism. We selected GB54, a triploid strain of the yeast species *Brettanomyces bruxellensis* with a 0.7% heterozygosity level. GB54 was sequenced by Oxford Nanopore long-read sequencing and Illumina short-read sequencing, then processed through the nPhase pipeline. Since we know that this strain is a triploid strain, we should expect a successful phasing to reflect that triploid nature by outputting three haplotypes per region. In our results, we observe that most regions have been phased into two or three haplotypes, with few small exceptions (Fig. 6).

**Fig. 7:**
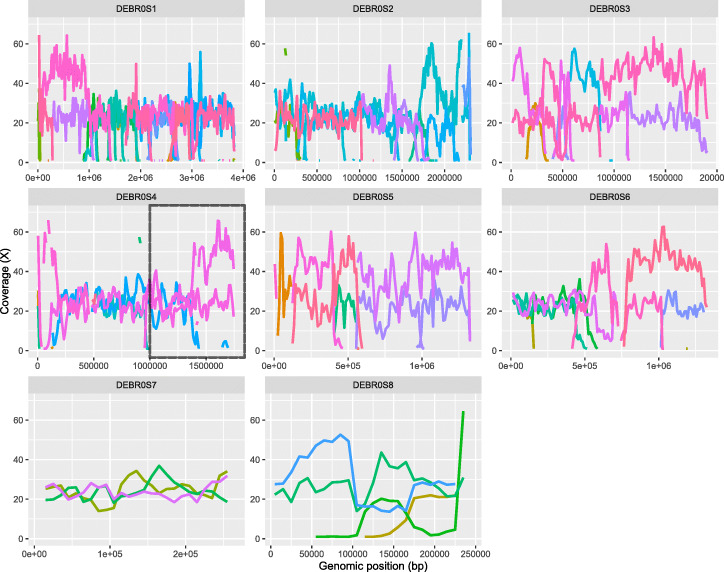
Coverage level of predicted haplotigs for the *Brettanomyces bruxellensis* strain. Each subgraph displays the coverage level of predicted haplotigs for a different chromosome of this 0.7% heterozygosity level triploid, the *Y* axis is the coverage level, and the *X* axis displays the position along the chromosome. All predicted haplotigs are color coded randomly as the ground truth is not known. We observe that in regions covered by only two haplotigs, one will be covered roughly twice as much as the other, whereas regions covered by three haplotigs tend to be equally covered

The regions that output more or less than three haplotypes are unexpected and potentially represent a phasing failure. For example, the highlighted region in Fig. 6, on chromosome 4, transitions from three haplotypes to only two haplotypes. By remapping each haplotig’s reads back to the reference and viewing the coverage, we note that regions phased into only two haplotigs have a coverage distribution consistent with the presence of only two haplotypes but three genomic copies (Fig. 7). One haplotig accounts for 2/3 of the coverage and the other haplotig accounts for the remaining 1/3 of the coverage. In Fig. 7, we highlighted the previously described region of chromosome 4, showing us that the three haplotigs in the first triploid region have roughly equal coverage, and in the region with only two haplotigs, one of them is twice as covered as the other.

**Fig. 8:**
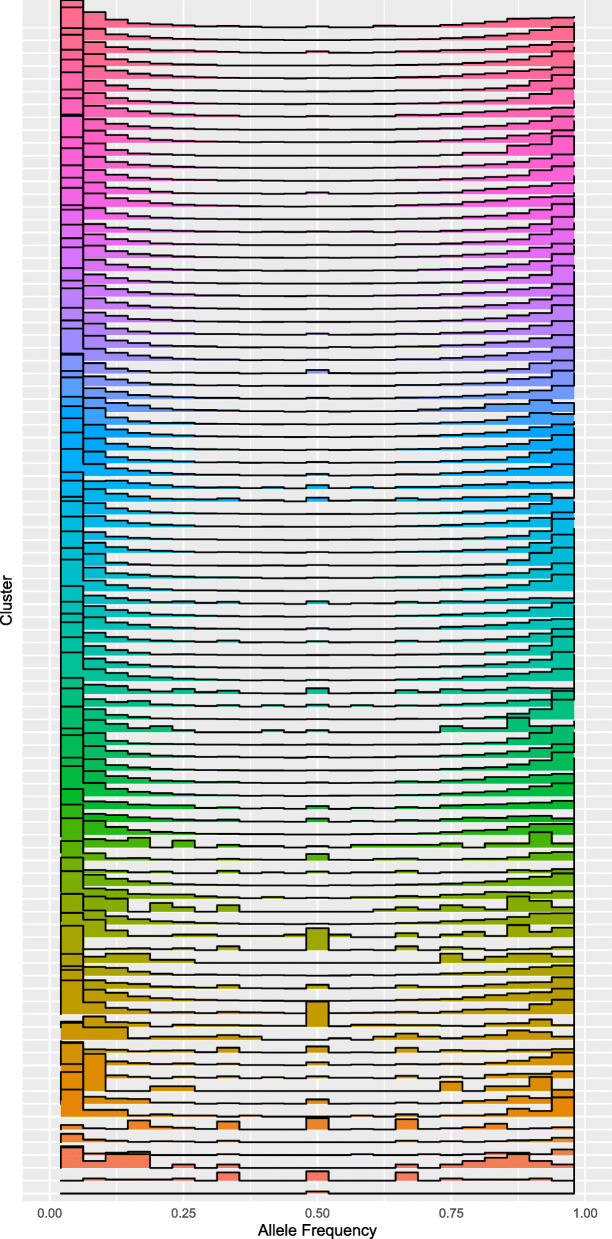
Allele frequency distribution of predicted haplotigs for the *Brettanomyces bruxellensis* strain. Each line displays the allele frequency distribution of predicted haplotig clusters. The height of each bar is the relative proportion of all heterozygous SNPs in that cluster and the *X* axis displays the allele frequency. All predicted haplotig clusters are color coded randomly as the ground truth is not known. We identify two clusters as having a heterozygous SNPs with a slightly high proportion of allele frequencies around 50%, one in green and one in orange towards the bottom of the figure

The haplotigs that represent 2/3 of the reads in the region they cover either represent one single haplotype which is present in two copies, or they represent two very similar haplotypes that were erroneously clustered into one by nPhase. By looking at the distribution of the heterozygous allele frequency within each haplotig’s corresponding cluster of reads, we show that few clusters are clearly enriched in allele frequencies around 50% (Fig. 8). Two of these clusters correspond to regions erroneously predicted to contain only one haplotig (Additional file 2: Fig. S2), confirming that the allele frequency within a haplotig cluster can reveal chimeric clusters. The absence of a noticeable enrichment in the allele frequencies of other clusters is further evidence that the predictions made by nPhase are highly accurate.

### Implementing automated cleaning steps

We observed in our initial in silico results that nPhase outputs many shorter haplotigs which consequently do not contain much phasing information. With our test on the *Brettanomyces bruxellensis* strain, we identified that we can use haplotig cluster allele frequency as a proxy for phasing quality. We also noted that, by design, nPhase will only output unique haplotypes which sometimes means that a region will be phased into fewer copies than might be naively expected based on ploidy. Finally, we also find that raw nPhase results can sometimes appear to be too fragmented.

To provide a method that begins addressing these issues, we developed a series of three steps intended to automatically clean nPhase’s raw results without significantly affecting accuracy:

1. Merging as many remaining haplotigs as possible together
2. Filtering out haplotigs that account for less than 1% of all coverage
3. Redistributing the reads of highly covered haplotigs

**Fig. 9:**
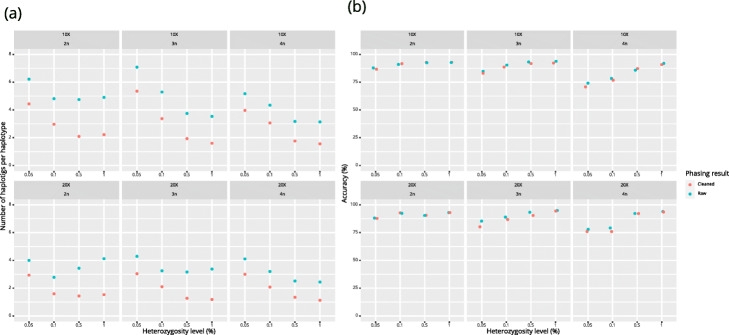
Contiguity and accuracy of nPhase results on virtual polyploids before and after automated cleaning. Each subgraph compares performance metrics for raw nPhase results with their automatically cleaned counterparts. Each point corresponds to a virtual genome of a given ploidy (n), coverage (X), and heterozygosity level. **a** We compare here the number of haplotigs per haplotype in all these conditions. We find a significant reduction in the number of haplotigs per haplotype for our cleaned results in all cases. **b** We compare here the accuracy (%) in all conditions. We find that the automated cleaning process has a small, negligible negative effect on accuracy in most cases, though not all

These steps are further described in the “Automated cleaning” section in the “Methods” section. We checked the effect of these steps on accuracy and number of haplotigs by applying them to the results of running nPhase with default parameters on all our virtual polyploids (10X and 20X coverage). Overall, our raw results had an average of 4.03 haplotigs per haplotype (Fig. 9a) and an average accuracy of 88.6% (Fig. 9b). After cleaning, we observed an average of 2.37 haplotigs per haplotype and an average accuracy of 87.4%. If we only consider our tests at 0.5% heterozygosity or higher, then our raw results had an average of 3.81 haplotigs per haplotype and an accuracy of 91.49%. After cleaning, we had an average of 1.87 haplotigs per haplotype with an accuracy of 91.44%.

**Fig. 10:**
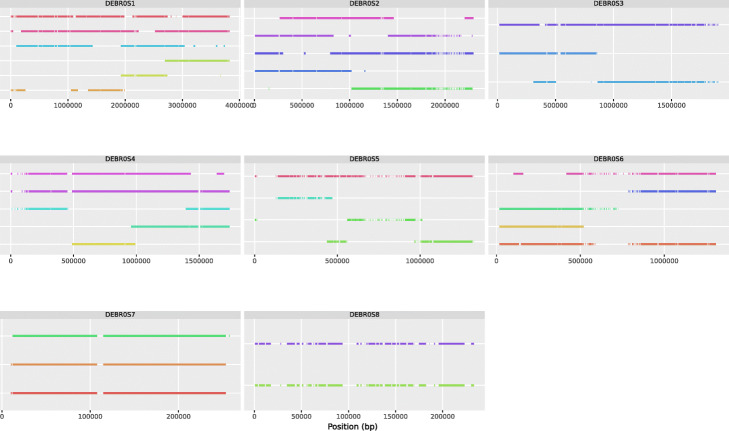
Automatically cleaned predicted haplotypes for the *Brettanomyces bruxellensis* strain. This figure represents an automatically cleaned version of Fig. 6. We note the presence of significantly fewer haplotigs, a higher contiguity, and the filling of the gap observed in the chromosome DEBR0S4. One notable change observed with the cleaned step is that chromosome 8 is predicted to have only two different haplotypes, which was not evident based on the raw results. For any sensitive application, it would be necessary to further scrutinize this prediction since the automated cleaning process is less rigorously validated

We tested this method on our results with GB54 and observe a significantly more contiguous phasing (Fig. 10). The cleaning process successfully redistributed the reads in the previously highlighted region of chromosome 4 (Additional file 2: Fig. S3) and merged haplotigs together in a way that renders the results much easier to interpret. The number of haplotigs has been reduced from 93 to 33, greatly reducing noise. We expect the accuracy not to have been negatively affected by this step based on the way the read coverage of cleaned haplotig clusters is distributed (1/3, 1/3, 1/3 coverage or 2/3, 1/3 coverage) and the allele frequency distributions of the cleaned haplotigs (Additional file 2: Fig. S4).

### Running the nPhase algorithm on chromosome 2 of the potato plant species *Solanum tuberosum*

We also tested nPhase on one chromosome of a larger, more repetitive plant genome. We used the autotetraploid *Solanum tuberosum* (potato) dataset generated for the WhatsHap polyphase paper [20]. We used the latest version of the DM1–3 516 R44 assembly as a reference (v6.1) [25]. We chose to limit this section to phasing chromosome 2. At 46 Mb, it is the shortest chromosome in the reference assembly (chromosome 1 is the largest with an 88 Mb chromosome), and is 30× larger than the longest chromosome in *S. cerevisiae* (chromosome 4, 1.5 Mb). We observe a 2.4% heterozygosity level for chromosome 2 based on Illumina data, more than twice as high as our most heterozygous test case (1%).

**Fig. 11:**
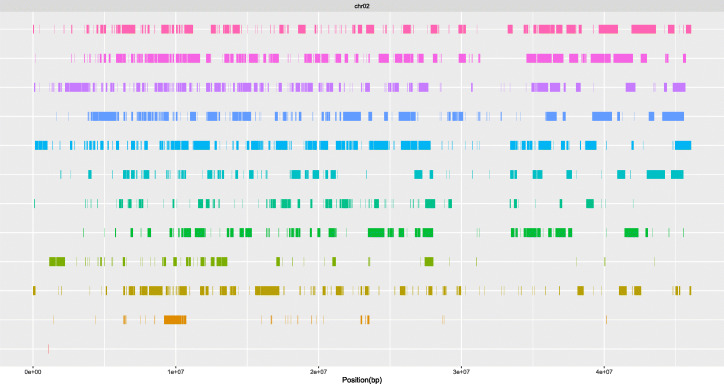
Automatically cleaned predicted haplotypes for the *Solanum tuberosum* strain. This figure represents automatically cleaned phasing results for chromosome 2 of *Solanum tuberosum*. 99% of phasing predictions are contained within the 12 largest haplotigs displayed here (the 12th is so sparse it is not visualized here). The cleaned result is significantly more contiguous than the raw 1129 haplotigs obtained directly from nPhase. Only around 16% of positions are covered by more than 4 haplotigs, likely in large part where one haplotig drops off in coverage while another starts increasing

To reduce computation time, we used a randomly sampled subset of heterozygous variants, effectively phasing at a 0.5% heterozygosity level. The raw results of nPhase yielded 1129 haplotigs, which were reduced to 25 haplotigs by a modified version of the automated cleaning steps; the final gap filling step was disabled in light of the fragmented nature of our raw results (Fig. 11). Of the 25 cleaned haplotigs output, we find 90% of predicted variants in the 9 largest cleaned haplotigs, and 99% of predicted variants in the 12 largest cleaned haplotigs. The remaining 13 cleaned haplotigs account for less than 0.6% of predictions made and could reasonably be filtered out.

We note that the haplotigs obtained skip around the chromosome, which may either be due to structural variation or to long reads being mapped in error to the wrong repetitive regions, thereby giving the illusion of widespread structural variation. We also checked the phasing predictions for the 5 longest genes of chromosome 2 (Additional file 2: Fig. S5) and found that they are coherent with our expectations for an autotetraploid.

## Discussion

We developed nPhase, an algorithm that relies on a few intuitive rules to process an input dataset of long reads, reduced to heterozygous positions, outputting as few clusters as possible which we have shown correspond to the true haplotypes with > 90% accuracy. By not specifying the ploidy of the sample in any step, we allow nPhase to adapt to the particularities of the dataset and do not run the risk of forcing an incorrect result to fit such an arbitrary algorithmic constraint. We provide nPhase as part of a pipeline that enables anyone to use their short- and long-read sequences of the same sample as inputs and obtain a list of SNPs and a fastQ file for each predicted haplotig.

Through our validation tests, we determined that there is a set of parameters for nPhase that performs optimally in nearly all of our test cases and that the algorithm performs well even with very low levels of genetic distance between haplotypes. We found that as little as 10X coverage can yield satisfying results. More complex cases, such as when there is a high ploidy coupled with a low heterozygosity, should benefit from higher coverage and a more stringent parameter for the minimum overlap (0.25 for example). Further investigation would be needed in order to more adequately define how these difficult samples should be treated. We also demonstrated with our benchmarking tests that nPhase outputs far more accurate and contiguous haplotigs than alternative polyploid phasing methods, and that this contiguity can be greatly improved at a very small cost to accuracy by using our simple automated cleaning process.

By testing nPhase on a triploid strain of the yeast species *Brettanomyces bruxellensis*, we were able to demonstrate that our method can be used on a real polyploid sample and provides a previously inaccessible insight into its haplotypic composition. We were able to show through the chosen sample the usefulness of a ploidy agnostic method which can adapt to a genome with a variable number of haplotypes. In the process of validating this real test case, we established two ways of qualitatively assessing phasing quality: checking the coverage plots of re-mapped haplotig reads and checking the allele frequency of reads that comprise a cluster. Our automated cleaning process yielded satisfying results, visibly improving the contiguity of the phasing, reducing noise, and filling some of the gaps observed in the raw phasing results.

We also tested nPhase on a much larger example, the 44 Mb long chromosome 2 of the potato plant species *Solanum tuberosum*, 30 times longer than the longest chromosome of *Saccharomyces cerevisiae*. This is a highly heterozygous autotetraploid with a highly repetitive genome and represents an important test case for nPhase. Despite the significantly increased complexity, we found that by using nPhase coupled with our cleaning steps we were able to produce a remarkably contiguous phasing prediction using only a fraction of the available data. The genes in particular we expect to be correctly phased, while we did not test the effects of nPhase on a highly repetitive test case with a known ground truth, limiting our certainty that the phasing was equally accurate in repetitive regions of the genome. In our analysis we did not take any extra steps to address the repetitive nature of the potato genome: we used a reference in which the repeats were not masked; we used all mapped reads, including those with low mapQ scores; and we did not check if variants called by short reads were reflected in the long reads. These are a few areas in which steps can be taken to improve the quality of the phasing. We also only used a fraction of all available heterozygous positions, and devoting more computing power and time to exploit more of that very relevant phasing information would presumably yield even better results.

As an alignment-based phasing algorithm, the performance of nPhase is going to be highly dependent on read length and the quality of the reference genome being mapped against. Consequently, structural variants between the sample and the reference, or even structural variants within the sample, are presently not explicitly identified and phased by the algorithm. In order to resolve structural variants between the sample and the reference or even between haplotypes in the sample, we need to rely on the information in split reads. Here, we used a simple strategy to stitch together some of the haplotigs we obtain without using all of the information contained within split reads. Leveraging the full potential of split reads is a crucial next step to improve the contiguity of phased blocks. The main difficulty in using split reads appears to be that these alignments are significantly less reliable and will need to be processed differently to account for that.

We made the choice not to base our phased blocks on insertion or deletion information. This information can still be obtained in the phased blocks by generating a de novo assembly using nPhase’s fastQ output for the relevant haplotig and could be integrated in future developments.

The rarity of raw accuracy numbers in the polyploid phasing literature derives from the observation that a single well-placed haplotype switching error has the potential to reduce the accuracy of a phased block by half. This led to the widespread adoption of using the SWitch Error Rate (SWER) or Vector Error Rate (VER) as the performance metric by which to compare polyploid phasing methods. This metric is only relevant to methods that accept the inevitability of switch errors, for which the raw measurement of the accuracy of predictions will not speak by itself. nPhase has achieved a very high level of accuracy (> 95%) and contiguity (1.25 haplotigs per haplotype) across most of our validation tests (in particular where the heterozygosity rate is of 0.5% of higher). The principal interest of providing performance metrics is to make it easy to assess the trustworthiness of a method’s results. For these reasons, we did not include the SWER in our performance metrics.

With the nPhase algorithm, we believe that the problem of switch errors in polyploid phasing is largely solved, the next important hurdle for polyploid phasing is finding an appropriate way to handle split reads to solve the remaining problems of contiguity and structural variants both within a sample and between the sample and the reference we align to. nPhase can still be used as a preprocessing step for any study of phased polyploid SVs and INDELs since that information is partially held within its output of fastQ files of phased reads.

Overall, nPhase provides, for the first time, an accurate and contiguous picture of polyploid genomes using only a reference genome and short and long reads. It paves the way for a better understanding of the origins of hybrid polyploid organisms, the true diversity of polyploid populations with potential hints on their origins and their relation to other diploid or haploid strains, and provides a clearer picture to investigate phenotypic effects tied to alleles which were previously inaccessible to us.

## Methods

### Total DNA extraction

Single colonies of each natural isolate were isolated by streaking on YPD media, containing ampicillin (50 μg/mL). Cells from one colony of each isolate were grown in 60 mL of YPD at 30 °C for 24 h. We extracted the total DNA of each isolate using the QIAGEN Genomic-tip 100/G kit, according to manufacturer’s instructions.

### Library preparation and sequencing

The kit NEBNext Ultra™ II DNA Library Prep Kit (Ipswich, USA) for Illumina (San Diego, USA) was used for short-read library preparation of the GB54 *Brettanomyces bruxellensis* isolate. The sample was sequenced on a single lane of NextSeq (Illumina) at the European Molecular Laboratory (EMBL) in Heidelberg, Germany. The strategy of sequencing was 75 paired-end (75PE).

For long-read sequencing, we used the EXP-NBD104 native barcoding kit (Oxford Nanopore) and the protocol provided by the manufacturer to barcode the total DNA of each of the isolates. The barcoded DNA was then quantified with a Qubit® 1.0 fluorometer (Thermo Fisher) and pooled together with an equal amount of DNA coming from each isolate. We then used the SQK-LSK109 ligation sequencing kit (Oxford Nanopore) to finish the library preparation. Finally, the library was loaded to a R9.3 flow cell for a 72-h run.

### Data pre-processing

The short reads are mapped to a reference genome using bwa [26] with the command bwa mem -M. We ran GATK [27] MarkDuplicates then variant called with GATK 4.0’s HaplotypeCaller --ploidy 2 to identify heterozygous positions. Long reads are basecalled, adapter trimmed, and demultiplexed by Guppy. They are then mapped to the same reference using NGMLR [28]. We keep only primary alignments and split reads with the samtools [29] flag 260.

We determine the positions of heterozygous SNPs from the VCF obtained by GATK by looking for positions where AF = 1.00 in the file. We reduced each long read to the set of variable positions it overlaps (Additional file 2: Fig. S6a). To simplify later computational steps, we remove long reads that are subsets of other long reads.

nPhase is only capable of phasing SNPs if they are identified by the variant calling step. This is not necessarily always the case, and the accuracy metrics are based on the total number of SNPs identified in the polyploid sample by the variant calling step. However, unidentified SNPs will still exist in the reads, so if the algorithm performs a proper clustering of the reads, the information will still be available and readily extracted by a closer view of the results.

### Context coverage

Long reads are error-prone, but it is important not to perform any form of error correction to ensure that the heterozygosity is not incorrectly flattened or mis-assigned. The nPhase pipeline works with raw long reads. In order to minimize the influence of these errors, we consider that SNP coverage is a useful indicator of quality. We count the number of times each heterozygous SNP is present in a specific context in our dataset. We define context as being the closest flanking heterozygous SNPs (two heterozygous SNPs upstream and two heterozygous SNPs downstream). The context information will be used to better inform the nPhase algorithm and allow it to escape the situation where a sequencing error randomly converts a well-supported SNP to another SNP that is well-supported in another haplotype (Additional file 2: Fig. S6b).

### Output results

Once nPhase is done running it outputs several files:

(i). A fastQ file for each haplotig containing all of the reads that have been clustered together for this haplotig, this file can then be used with a de novo assembly or alignment tool for further analysis.
(ii). A tab separated file listing the consensus base for each heterozygous position contained within each haplotig. There are three columns: chromosome, position, and consensus base. If two different bases are equally represented for a given position and equally well supported within the cluster, they will both be represented in this file on separate lines. This file is sorted by position.
(iii). A plot representing the different haplotigs along the reference genome, similar to the one displayed in Fig. 3 but lacking the haplotype color code since the ground truth is not known in a typical use case of nPhase.

### nPhase parameter description

nPhase has a total of 4 parameters which can be adjusted to better fit the sample. These parameters are the following (Additional file 2: Fig. S7):

#### *S*, the minimum fraction of similarity between two reads

When two reads overlap with each other, we calculate their similarity by dividing the number of heterozygous SNPs they share by the number of heterozygous positions they both cover. If that fraction is smaller than the parameter *S*, then we will consider that these two reads cannot be part of the same haplotype. This parameter can be set to any fraction between 0 and 1, by default it is set at 0.01, or 1% similarity.

#### *O*, the minimum fraction of overlap between two reads

When two reads overlap with each other, we can count the number of heterozygous positions they both cover. If they both cover more than 100 heterozygous positions, this parameter is ignored. If they cover fewer than 100 heterozygous positions, then we calculate the overlap by dividing the number of heterozygous positions the two reads have in common by the total number of heterozygous positions covered by the smaller of the two reads. In this case, smaller does not necessarily mean a shorter read; it means a read that covers fewer heterozygous positions. If this overlap is smaller than the parameter *O*, then we consider that these two reads do not overlap enough for us to conclusively determine if they are part of the same haplotype. This parameter can be set to any fraction between 0 and 1; by default, it is set at 0.1, or 10% overlap.

#### *L*, the minimum number of reads supporting a haplotig

Once nPhase has clustered all of the reads into different haplotigs, the user may want to filter out all haplotigs that are supported by fewer than *N* reads. This parameter can be set to any integer *N* ≥ 0, by default it is set at 0. If set to *N*, it will not output any cluster supported by fewer than *N* reads.

#### ID, the maximum amount of change when merging clusters

When nPhase considers merging two clusters of reads into one new cluster, it must determine if these two clusters are similar enough to warrant merging them together or if they should remain unique clusters, representative of unique haplotypes. Since these are clusters, every heterozygous position is potentially covered multiple times, sometimes with different reads in the same cluster indicating conflicting bases for the same position. We can calculate the number of reads voting for each base in a given cluster and determine the “demographics” for that position. We can take this further and have an overview of every heterozygous position in the cluster and how well-supported each base is. The base that has the majority of support is considered to be the “true” base for that cluster. When we merge two clusters together, we potentially change these “demographics”. These changes either further strengthen the position of the majority base for a given position, in which case there is no negative change in the cluster’s “identity” or they weaken the majority base’s position and cause a negative change to the cluster’s “identity”. When there are negative changes to the cluster’s “identity” we can calculate the amount of change that has occurred and if that amount is too high the clusters are not allowed to merge. This parameter can be set to any fraction between 0 and 1, by default it is set at 0.05, or a 5% ID change tolerance.

These parameters are set by default, though they can be modified if needed. The nPhase algorithm will use these parameters as limitations to determine which reads it is allowed to cluster together into haplotigs and which clusters of reads it can merge together into longer haplotigs. Ideally, only the ID parameter needs to be modified, keeping all other parameters very low and forcing the algorithm to merge clusters as aggressively as allowed by the ID parameter.

### Identifying optimal parameters

In order to determine which parameters nPhase should use by default, it is important to understand how these parameters affect the results. Ideally, we will find that there is a set of parameters which is optimal for all possible combinations of ploidy and heterozygosity level; such a set would then become the default recommended parameters for nPhase. If no such set of parameters appears to exist, the next best case is to minimize the impact of as many of the available parameters as possible in order to reduce the parameter a user would need to explore when using nPhase to phase their dataset.

Through our tests, we find that there is a narrow range in the parameter space that results in the optimal performance of nPhase. Intuitively, the optimal strategy appears to be to set the minimum similarity and minimum overlap parameters down to a low value so that all of the reads in the dataset are allowed to merge into a cluster, and to only worry about finding an appropriate threshold for the ID change parameter. Since the ID change parameter controls how dissimilar two clusters need to be in order to be considered two different haplotypes, it is fitting for this parameter alone to have the most pronounced impact on the quality of results. If set too low, nPhase will consider small sequencing errors to be evidence of alternate haplotypes, and if set too high, it will allow different haplotypes to merge into chimeric and wrong results.

To demonstrate this, we ran nPhase 125 times on 24 different samples of varying coverage, ploidy, and number of heterozygous SNPs for a total of 3000 tests. These 125 tests represent every possible combination of the minimum similarity *S*, minimum overlap *O*, and maximum identity change ID parameters for the following values: 0.01, 0.05, 0.1, 0.15, 0.25.

The *L* parameter was set to 0 for these tests since it is intended for use to clean up results by removing small, lowly supported haplotigs and we wanted to determine how nPhase performs without throwing away any of the data.

We found that *S*, the minimum similarity parameter, had no influence on the results at these levels (Additional file 2: Fig. S8a). *O*, the minimum overlap parameter, needs to be at least at 0.1 and seems to show very minor improvements in accuracy at higher levels (Additional file 2: Fig. S8b). The ID parameter has the most influence on the accuracy of the results, with values of 0.05 and 0.1 yielding the best results (Additional file 2: Fig. S8c).

We then looked at the effects of *O* and ID on the average number of haplotigs per chromosome per parent. We found that the number of haplotigs slightly increases with *O* (Additional file 2: Fig. S9a), while ID has a strong effect on the contiguity of the results (Additional file 2: Fig. S9b). A higher value for ID leads to a more contiguous assembly, though this comes at the cost of accuracy (Additional file 2: Fig. S9c). We again find that values held between 0.05 and 0.1 provide good results. If we separate our tests by ploidy, we can see that, as the ploidy increases, the optimal choice for the ID parameter narrows down around 0.05 (Additional file 2: Fig. S10).

Based on our tests, we find that the following set of parameters is the best adapted to handle any sample: *S* = 0.01, *O* = 0.1, *L* = 0, ID = 0.05. We use these as our default parameters.

### Influence of coverage

We sought to establish the effects of coverage on the quality metrics of nPhase’s predictions. To do so, we performed our tests on a 10X per haplotype dataset and a 20X per haplotype dataset. We found that both accuracy and contiguity are improved by the higher coverage level of 20X per haplotype (Additional file 2: Fig. S11). This effect is observed across ploidy and heterozygosity levels, though the accuracy effects are more pronounced for higher ploidy, lower heterozygosity level samples.

A low number of haplotigs per haplotype is not always a good sign of high contiguity as it can be compatible with a high rate of chimeric haplotigs. Therefore, we looked at the contiguity effects of coverage for our tests using default parameters, which we have previously determined output accurate results. Based on these tests, we were able to confirm that the 20X dataset is more contiguous than the 10X dataset (Additional file 2: Fig. S11b). We therefore used the 20X datasets as part of our default analysis.

### Split read stitching step

Some reads align to two or more very distant sequences in the reference genome. These reads can represent a structural variation between the sample and the reference being mapped to. We split them into the different segments that align to the reference and refer to them to as split reads.

Split reads can be very misleading and trusting them blindly would result in chimeric haplotigs. We developed a simple pre-processing strategy to integrate part of the information contained by these split reads.

We run nPhase a first time to obtain our initial haplotigs. We expect some of the edges of haplotigs to correspond to structural variants such as inversions or large INDELs so we identify the SNPs at the edges of these clusters. These are the SNPs which we expect to be included in the split reads that can connect two haplotigs separated by a structural variant, so they are the most trustworthy SNPs in our split read dataset. We reduce each split read to only the heterozygous SNPs that overlap with these regions and re-run the nPhase algorithm with these reads included. Clusters are currently not allowed to combine reads from different reference chromosomes, so split reads can only be used to improve the contiguity of haplotigs on the same reference chromosome.

As described, nPhase does not exploit the information contained in split reads to the fullest extent, only attempting to improve contiguity by stitching together haplotigs on the same chromosome. Once there are only a few remaining haplotigs, further improving contiguity necessarily means stitching longer haplotigs together. This presents a very real danger of creating chimeric haplotigs that have very strong negative effects on accuracy. To validate the usefulness of these steps and this method of using the split read data, we ran 3000 tests of nPhase both with split read information and 3000 tests without in order to determine the effects of our split read stitching strategy on both contiguity and accuracy. We found that the contiguity did significantly improve across all of our tests that included split read information, compared to those that did not (Additional file 2: Fig. S12a). Encouragingly, when comparing the accuracy distributions of the two sets of tests, they are virtually identical (Additional file 2: Fig. S12b). The tests that used split reads were very slightly less accurate than their counterparts but much more contiguous, motivating our decision to integrate the use of split read information in nPhase.

### Automated cleaning

We established a three-step automated cleaning procedure to quickly reduce noise and improve the contiguity of raw nPhase results. These steps have a negligible negative effect on accuracy in our in silico tests while greatly reducing the number of haplotigs per haplotype.

The first step of the automated cleaning process is the merging step. We first calculate, for every raw cluster output by nPhase, the proportion of bases that disagree with the consensus base. We call this the discordance level of the cluster, and it is equivalent to the summed minor allele frequencies represented in this cluster. We calculate the mean level of discordance across all clusters output by nPhase and we use this number as our stopping point. Our goal is to find pairs of clusters that merge together without increasing our risk of merging two different haplotypes into one. For each pair of clusters, we calculate the discordance level that we would obtain if we merged them together. If that discordance level is lower than the average discordance level calculated previously, then we can allow the merge to occur. If it is higher, then we do not allow the merge. Once there are no pairs of clusters left that are allowed to merge, we end this step.

The second step is a filtering step. We sort all remaining clusters by the total coverage they represent, and we only remove clusters that account for the smallest 1% of coverage. This allows us to get rid of the small noisy clusters we have observed in our results.

The third and final step is the filling of gaps. We calculate, for each chromosome, the average coverage level of all the haplotigs (each haplotig counts as 1X; we are not looking at the coverage level of the reads contained within the clusters that define the haplotigs). We round this coverage level, and if it is rounded up to *n* (from 2.6 to 3 for example), then we identify the regions of the chromosome that are covered less than *n* times. For each such region, we identify the most covered cluster, split its reads in half, and redistribute them such that we have effectively filled the gap. This step presumes that the gap is due to a large region of the chromosome having the same haplotype in at least two copies of the genome (as evidenced by the coverage level being twice as high, for example).

We validated these steps by running them on our virtual polyploids and comparing the accuracy and contiguity results to the raw nPhase results.

### Performance limits

With default parameters, the nPhase algorithm took between 1 min and nearly 5 h of runtime on a single CPU (the nPhase algorithm has not been parallelized), and between 0.6 GB and 31.8 GB of memory (Additional file 1: Table S3). The runtime and memory usage are clearly tied to the ploidy and heterozygosity level. A higher ploidy and higher heterozygosity level translates to a significant increase in runtime and memory usage. Each diploid test, up to 1% heterozygosity, ran in less than an hour and 10 min and used less than 8 GB of memory. Triploid tests took a minimum of 3.5 min of CPU time and 0.9 GB of memory to run for the 0.05% heterozygosity level example, and a maximum of 3 h and 10 min of CPU time and 19 GB of memory for the 1% heterozygosity level test. The tetraploid examples were the most resource intensive, using up a minimum of 6 min of CPU time and 1.25 GB of memory for the 0.05% heterozygosity level, and a maximum of 4 h and 50 min of CPU time and 31.8 GB of memory to run. nPhase can output results in a reasonable time using moderate memory resources. If run on a particularly large genome in a time-sensitive context, nPhase could be applied to individual chromosomes in parallel. It is also reasonable to consider down-sampling the number of SNPs to a heterozygosity level of around 0.5% given the results obtained are comparable and run in less than half the time as the 1% heterozygosity level tests. All of the heterozygous SNPs would still be present in the long reads and could be recovered from the fastQ files associated to the predicted haplotypes.

### Assessing the quality of the *Brettanomyces bruxellensis* phasing

We used the nPhase pipeline to phase a triploid *Brettanomyces bruxellensis* strain. nPhase predicted that a number of regions had three haplotypes and that others had only two. In order to verify the accuracy of these predictions, we visualized our data in two complementary ways.

First, we checked the coverage levels of the predicted haplotigs. We mapped the fastQ files generated by nPhase back to the same reference genome, then generated coverage plots using a 5-kb window (the genome is 13 Mb long). In order to minimize the visual noise in Fig. 7, we only displayed the longest haplotigs that account for 90% of all coverage.

Second, we checked the allele frequency distribution within each cluster. We cross-referenced the file generated by nPhase in the VariantCalls/LongReads folder containing a list of reads and the identity of every base at each heterozygous position with the file generated in the Phased folder containing a list of the final haplotig clusters and the list of reads that comprise them. Using both files, we were able to determine the allele frequency for each position within every haplotig cluster. We selected only the positions covered by at least 20 reads to generate Fig. 8.

### Phasing an autotetraploid strain of *Solanum tuberosum* with nPhase

We obtained whole genome Oxford nanopore and Illumina read data for an autotetraploid strain of *Solanum tuberosum* from the WhatsHap polyphase paper under accession number PRJEB39456. We used v6.1 of the DM1–3 516 R44 assembly as a reference, selecting the version without any repeat masking. We mapped all the reads to the full genome, but we then extracted all of the reads which mapped to chromosome 2 to run the phasing algorithm on. Of the 2.4% of heterozygous positions observed in chromosome 2 according to variant calling on Illumina data, we only kept a randomly sampled 0.5% for the phasing in order to save calculation time.

Once we obtained raw nPhase results, we ran our automated cleaning steps in order to improve contiguity and reduce the complexity of our results.

## Supplementary Information

**Additional file 1. **This file is an excel spreadsheet containing all of the supplementary tables. **Table S1** - *Saccharomyces cerevisiae* strains used in our validation experiment. **Table S2** - Results of running nPhase on our validation dataset using the optimal parameters identified. **Table S**3 - Performance metrics of running nPhase on that validation dataset.

**Additional file 2. **This file is a word document containing all of the supplementary figures. **Fig. S1** Raw nPhase results for the different simulated polyploids in the validation dataset. **Fig. S2** Coverage levels of chimeric haplotigs. **Fig. S3** Coverage levels of all haplotigs in the cleaned *Brettanomyces bruxellensis* phasing results. **Fig. S4** Frequency distribution of alleles in the haplotig read clusters for the cleaned *Brettanomyces bruxellensis* phasing results. **Fig. S5** nPhase phasing results for the 5 longest genes in the *Solanum tuberosum* annotation. **Fig. S6** Long read pre-processing steps reducing them to sequences of variable positions. **Fig. S7** Different parameters used in nPhase. **Fig. S8** Effects on accuracy of running nPhase using different parameter combinations. **Fig. S9** Effects on contiguity of running nPhase using different parameter combinations. **Fig. S10** Effects of ploidy on the optimal choice of the ID parameter. **Fig. S11** Effect of coverage on accuracy and contiguity. **Fig. S12** Effects of including split reads on accuracy and contiguity.

**Additional file 3 Review history.**

## Data Availability

The nPhase algorithm and the nPhase pipeline are both available under the open source GNU General Public License v3.0 at https://github.com/OmarOakheart/nPhase [30]. Oxford Nanopore sequencing data is available under the study accession number PRJEB39456. Illumina short-read data for the *Saccharomyces cerevisiae* strains are taken from the 1011 yeast genomes project [24] and their SRA accessions are the following: ERR1308732 [31], ERR1309429 [31], ERR1308952 [31], and ERR1308675 [31]. Illumina and Oxford Nanopore data for the *Brettanomyces bruxellensis* GB54 strain is available under the study accession number PRJEB40511. Illumina and Oxford Nanopore data for the *Solanum tuberosum* strain used is available under the study accession number PRJNA587397 [32].
