# Supplementary material for: nPhase: an accurate and contiguous phasing method for polyploids
Source: Genome Biol. 2021 Apr 29;22:126. doi: 10.1186/s13059-021-02342-x (PMC8082856; doi:10.1186/s13059-021-02342-x)
Supplement: Supplementary file 3 — Additional file 3 Review history. [file 13059_2021_2342_MOESM3_ESM.docx]

**1^st^ round**

**Reviewer 1**

The manuscript describes nPhase, an approach to phase polyploid genomes. The algorithm is discussed clearly and thoroughly evaluated. The approach performs well on simulated data of limited complexity (based on yeast genomes), less so on a real-life triploid genome. As the algorithm is relatively straightforward compared to existing methods, the major advantage seems to be the use of a combination of short and long reads. Existing methods that exploit long reads struggle to accurately detect variant positions, which is circumvented in the nPhase approach using the short reads.

Suggested revisions:

1. The text should reflect the fact that the main reason for good performance of the approach is most likely the combination of long and short reads, rather than the novelty of the algorithm; e.g. in the Introduction (p3 from line 25), the first paragraph of the Discussion (p12 from line 28) and the sixth paragraph (p13 from line 46). It should also be made clear that WhatsHap does not use short reads, which makes the comparison less straightforward than it currently seems (p10, from line 7; Fig. 5, caption).

2. To better assess the performance improvement in terms of the algorithm, it would be highly informative (and not much work) to apply WhatsHap polyphase to long reads cleaned using short reads (by any of the methods discussed in https://www.biorxiv.org/content/10.1101/519330v2.full.pdf).

3. There is a reason why WhatsHap and other tools report performance in terms of switch error: one switch can lead to many erroneous calls. Please report switch error rate as well in the comparison, next to the currently reported measures.

4. Please discuss why, in Figure 3, in numerous places overlapping haplotigs are not (or cannot be) merged.

5. The experiment on the real triploid sample (GB54) is discussed extensively in terms of coverage, but seems highly fragmented (in particular for the first chromosomes); please discuss the potential reasons for this. Have the same settings been used for phasing this triploid as for the simulated cases?

6. The yeast genomes used are relatively simple compared to those of polyploid plants, in which there is a lot of interest. It would be good to discuss the applicability of nPhase to plant genomes, which are typically far larger (runtime feasible?) and contain many repeats (influencing the clustering step?). Both genome size and repeat content could of course also be evaluated by simulation.

**Reviewer 2**

The authors of the manuscript "nPhase: An accurate and contiguous phasing method for polyploids" describe a new phasing method that focuses on phasing variations over a polyploid genome. It is quite interesting as nPhase does not seem to require a polyploidy level estimation in contrast to its main competitor WhatsHapp. Overall the manuscript is in some sections a bit hard to follow, but gives great details about the benchmarks. I would also encourage the authors to think of applications outside of yeast, but i understand that its the main target here. In the following I list my questions and concerns in no particular order:

1. How do you report the different polyploidies in the VCF file ? To be precise phase blocks don’t work anymore for polyploid genomes as certain haplotypes might be only partially phased.

2. What is the input? A VCF file with mapped reads to a reference, or really raw reads only? This is not clearly stated.

3. Are you identifying the SNV or is that part of the pipeline?

4. How is the consensus calculated when merging a cluster ? Is only the one consensus sequence stored and thus a new read would mean a consensus of the previous consensus plus the new read? Or do you take some weights into account ? As far as I understood that is important for the decision to merge and if you take some weights the decision will change over time right ?

5. The haplotyigs per chromosome are a bit hard to follow. I understand that most correct it should be equal to the polyploidy level. However, un phased regions might be preferred, right ? Would you also report the un phased % of the genomes or haplotypes? I know this is tricky as there are no really standards on how to compare polyploid phasing results.

6. I would be also interested to see the runtime of nPhase.

7. As a minor comment: if your method is really independent of the number of polyploidy and clusters the reads together as I understood from the main comments: Why don't you try other plants to cancer ? The main disadvantage from Whatshapp as far I as understood is its dependency of knowing the polyploidy and keeping it fixed along the genome / chromosome. This way nPhase would show its uniqueness and advantage.

8. I know its against the main idea, but have you considered to ask the user for a polyploidy level estimate ? This could help in the post filtering/ananlysis of nPhase. You could think about this parameter as a suggestion. Or you estimate this based on the findings along a chromosome. This could help identifying if there are only 2 out of 3 haplotypes reported as the % identity drops and thus one cluster has a higher coverage than the other vs. a real phasing error.

**Authors’ response**

Reviewer #1: The manuscript describes nPhase, an approach to phase polyploid genomes. The algorithm is discussed clearly and thoroughly evaluated. The approach performs well on simulated data of limited complexity (based on yeast genomes), less so on a real-life triploid genome. As the algorithm is relatively straightforward compared to existing methods, the major advantage seems to be the use of a combination of short and long reads. Existing methods that exploit long reads struggle to accurately detect variant positions, which is circumvented in the nPhase approach using the short reads.

**1. The text should reflect the fact that the main reason for good performance of the approach is most likely the combination of long and short reads, rather than the novelty of the algorithm; e.g. in the Introduction (p3 from line 25), the first paragraph of the Discussion (p12 from line 28) and the sixth paragraph (p13 from line 46). It should also be made clear that WhatsHap does not use short reads, which makes the comparison less straightforward than it currently seems (p10, from line 7; Fig. 5, caption).**

[R] WhatsHap polyphase does use short reads, and in fact recommends it. From the whatshap guide (https://whatshap.readthedocs.io/en/latest/guide.html):

"The reads used for variant calling (to create the input VCF) do not need to be the same as the ones that are used for phasing. We recommend that high-quality short reads are used for variant calling and that the phasing is then done with long reads, see the recommended workflow."

We therefore maintain that the good performance of the approach is not exclusively due to the combination of long and short reads. When we compare nPhase to polyphase in our benchmark, we use the exact same inputs for both (long and short reads).

**2. To better assess the performance improvement in terms of the algorithm, it would be highly informative (and not much work) to apply WhatsHap polyphase to long reads cleaned using short reads (by any of the methods discussed in https://www.biorxiv.org/content/10.1101/519330v2.full.pdf).**

[R] Since WhatsHap polyphase does use short reads, this did not seem necessary (see previous comment).

**3. There is a reason why WhatsHap and other tools report performance in terms of switch error: one switch can lead to many erroneous calls. Please report switch error rate as well in the comparison, next to the currently reported measures.**

[R] One of the main selling points of nPhase is that the accuracy is high enough that we no longer need to explain very low accuracy numbers using the switch error metric. We hope to move the discussion of accuracy metrics in polyploid phasing away from obscure, hard to interpret metrics which hide very low accuracy results such as the switch error rate and the minor error correction in favor of universal metrics such as accuracy, error rate and false negative rate (described as missing rate in the paper). As the review mentions, a single switch error can have a devastating effect on accuracy. To illustrate with a simple example, comparing two methods on the basis of switch error doesn’t allow one to differentiate between a method that has 1 switch error in the middle of its haplotig which leads to 50% accuracy, and a method that has 1 switch error at the end of its haplotig, leading to 99% accuracy.

**4. Please discuss why, in Figure 3, in numerous places overlapping haplotigs are not (or cannot be) merged.**

[R] We agree that it does seem that such overlapping haplotigs should be merged. In the raw nPhase results, this simply means that merging the two haplotigs would have modified them by a number that exceeds the default maxID parameter we use. However, in a practical sense we would normally later manually join some of these haplotigs together. We developed and added a series of automated cleaning steps to nPhase and to the paper which attempt to do much of this manual post-processing work without a strong influence on accuracy.

**5. The experiment on the real triploid sample (GB54) is discussed extensively in terms of coverage, but seems highly fragmented (in particular for the first chromosomes); please discuss the potential reasons for this. Have the same settings been used for phasing this triploid as for the simulated cases?**

[R] The same settings have been used for this triploid as the simulated cases. Upon further investigation, we identified an error in the split read processing step which affected GB54 due to an issue between the naming convention that was selected for it and the code that was written. We confirmed that this error did not have an effect on our other tests, and re-ran nPhase on GB54, yielding more contiguous results. The issue persists, though to a much lesser degree. One possible explanation is that *Brettanomyces bruxellensis* strains are known to harbor many structural variants, another possibility is a combination of a lower mean read length (avg 11 kb for GB54 vs 15 kb for the virtual polyploids) and a lower mean baseQ score (avg 9.3 for GB54 vs 10.3 for the virtual polyploids, for reference, a baseQ of 9.3 is 88% accurate, and of 10.3 is 90% accurate). The automated cleaning step mentioned previously also does some work to reduce the discontinuity.

**6. The yeast genomes used are relatively simple compared to those of polyploid plants, in which there is a lot of interest. It would be good to discuss the applicability of nPhase to plant genomes, which are typically far larger (runtime feasible?) and contain many repeats (influencing the clustering step?). Both genome size and repeat content could of course also be evaluated by simulation.**

[R] We ran nPhase on chromosome 2 of the potato plant species *Solanum tuberosum* to test how nPhase performs on significantly larger test cases. This chromosome is 30x larger than the longest *S. cerevisiae* chromosome we tested nPhase on, and is a highly heterozygous, high repeat content autotetraploid. Genome size poses a surmountable challenge, and it seems clear that repeat content does have a significant effect that would be interesting to study further.

Reviewer #2: The authors of the manuscript "nPhase: An accurate and contiguous phasing method for polyploids" describe a new phasing method that focuses on phasing variations over a polyploid genome. It is quite interesting as nPhase does not seem to require a polyploidy level estimation in contrast to its main competitor WhatsHapp. Overall the manuscript is in some sections a bit hard to follow, but gives great details about the benchmarks. I would also encourage the authors to think of applications outside of yeast, but i understand that its the main target here. In the following I list my questions and concerns in no particular order:

**1. How do you report the different polyploidies in the VCF file ?  To be precise phase blocks don’t work anymore for polyploid genomes as certain haplotypes might be only partially phased.**

[R] We currently don't output a VCF file, opting instead to output a file which lists the SNPs that define each haplotig’s consensus sequence.

**2. What is the input? A VCF file with mapped reads to a reference, or really raw reads only? This is not clearly stated.**

[R] The input is raw short reads, raw long reads, and a reference sequence. It is described at the end of the introduction "The required inputs are a reference sequence as well as long and short read sequencing data." and at the beginning of the results section "We developed the nPhase pipeline, an alignment-based phasing method and associated algorithm that run using three inputs: highly accurate short reads, informative long reads and a reference sequence."

**3. Are you identifying the SNV or is that part of the pipeline?**

[R] The pipeline identifies SNPs (not indels or any structural variants), using GATK. Technically, we only use GATK to identify SNP positions, and then we take mapped long reads and look at which base is present for each long read. So, if the illumina data says there can only be a C or a T in a given position, but the long read says there is a G, we believe the long read.

**4. How is the consensus calculated when merging a cluster ? Is only the one consensus sequence stored and thus a new read would mean a consensus of the previous consensus plus the new read? Or do you take some weights into account ? As far as I understood that is important for the decision to merge and if you take some weights the decision will change over time right ?**

[R] The consensus is recalculated every time we merge clusters, so there are weights which can slowly change over successive merges. The ID change parameter measures how much the weights change with each merge, and blocks attempts to merge two clusters if the weights change too much, even if the result does not change the consensus.

**5. The haplotyigs per chromosome are a bit hard to follow. I understand that most correct it should be equal to the polyploidy level. However, un phased regions might be preferred, right ? Would you also report the un phased % of the genomes or haplotypes?  I know this is tricky as there are no really standards on how to compare polyploid phasing results.**

[R] Rather than reporting the number of haplotigs per chromosome, we reported the number of haplotigs per chromosome per haplotype. Meaning, the most correct it should be equal to 1. This requires us to know the true number of haplotypes, which was possible for the simulated example. It is not a perfect metric because it does not take into account the size of these haplotigs. Perhaps a more intuitive metric would be one that draws parallels with de novo assembly metrics. For example, the number of haplotigs needed to phase 90% of heterozygous variants (P90?). The ideal number would then be equal to the number of haplotypes. The advantage of number of haplotigs per chromosome is that it can be calculated even when we do not know the truth. Whereas calculating 90% of heterozygous variants implies we already know the ground truth.

**6. I would be also interested to see the runtime of nPhase.**

[R] The runtime of nPhase is given at the end of the Methods section, under "Performance limits" and in the supplemental material (supplemental table 3).

**7. As a minor comment: if your method is really independent of the number of polyploidy and clusters the reads together as I understood from the main comments: Why don't you try other plants to cancer ? The main disadvantage from Whatshapp as far I as understood is its dependency of knowing the polyploidy and keeping it fixed along the genome / chromosome. This way nPhase would show its uniqueness and advantage.**

[R] We ran nPhase on chromosome 2 of the potato plant species *Solanum tuberosum* to test how nPhase performs on significantly larger test cases. This chromosome is 30x larger than the longest *S. cerevisiae* chromosome we tested nPhase on, and is a highly heterozygous, high repeat content autotetraploid. While an interesting idea, we could not find a human cancer dataset with both long and short read full genome data and sufficient coverage. We would argue that the main disadvantage of WhatsHap polyphase is its lack of accuracy, which isn’t necessarily caused by its assumption of a fixed ploidy.

**8. I know its against the main idea, but have you considered to ask the user for a polyploidy level estimate ? This could help in the post filtering/ananlysis of nPhase. You could think about this parameter as a suggestion. Or you estimate this based on the findings along a chromosome. This could help identifying if there are only 2 out of 3 haplotypes reported as the % identity drops and thus one cluster has a higher coverage than the other vs. a real phasing error.**

[R] Algorithmically, there isn't an easy way to introduce a way to coerce a specific ploidy into nPhase, and as we see with the real triploid example, the presence of three different copies of a genome doesn't necessarily mean the presence of three different haplotypes across its genome. Though, it is clear that nPhase would greatly benefit from an efficient post-processing method as the raw results take a lot of manual work to clean up. We have developed a cleaning method for nPhase, mainly based on the concept of minimizing cluster discordance (the proportion of bases that disagree with the consensus base) after the first step. It is now detailed in the manuscript. It does not take user input, and does not attempt to estimate the number of haplotypes based on clustering results. It simply iteratively merges clusters together and checks each time if the discordance value is significantly higher. It determines which discordance values are acceptable by calculating the discordance values of the initial phasing results (which we believe are highly accurate). Then it tries to minimize the total discordance level of all clusters by merging together those that overlap.

**2^nd^ round**

**Reviewer 1**

I stand corrected on my original points #1 and #2. The authors addressed most comments satisfactorily, in particular by adding an experimental evaluation on a plant chromosome.

Comment #3 is the exception. I do not consider switch error hard to interpret, but rather providing a different perspective, i.e. whether inaccuracies occur spread over the genome or due to one (or a few) switches. Incorporating this measure - next to accuracy, as I suggested, so not hiding anything - would give users potentially useful information on the comparative merits of different methods. My suggestion would still be to report these numbers.
